# Matcha Green Tea Alleviates Non-Alcoholic Fatty Liver Disease in High-Fat Diet-Induced Obese Mice by Regulating Lipid Metabolism and Inflammatory Responses

**DOI:** 10.3390/nu13061950

**Published:** 2021-06-06

**Authors:** Jihong Zhou, Yueer Yu, Lejia Ding, Ping Xu, Yuefei Wang

**Affiliations:** Tea Research Institute, Zhejiang University, Hangzhou 310058, China; zhoujihong@zju.edu.cn (J.Z.); 3160100238@zju.edu.cn (Y.Y.); 3170100613@zju.edu.cn (L.D.); zdxp@zju.edu.cn (P.X.)

**Keywords:** matcha green tea, nonalcoholic fatty liver disease, nonalcoholic steatohepatitis, fatty toxicity, lipid droplets-associated proteins, cytochrome P450

## Abstract

Lately, matcha green tea has gained popularity as a beverage and food additive. It has proved to be effective in preventing obesity and related metabolic syndromes. However, the underlying mechanisms of its control effects against non-alcoholic fatty liver disease (NAFLD) are complicated and remain elusive. In the present study, we performed an in vivo experiment using male C57BL/6 mice fed with a high-fat diet and simultaneously treated with matcha for six weeks. Serum biochemical parameters, histological changes, lipid accumulation, inflammatory cytokines, and relevant indicators were examined. Dietary supplementation of matcha effectively prevented excessive accumulation of visceral and hepatic lipid, elevated blood glucose, dyslipidemia, abnormal liver function, and steatosis hepatitis. RNA sequencing analyses of differentially expressed genes in liver samples indicated that matcha treatment decreased the activity of lipid droplet-associated proteins and increased the activity of cytochrome P450 enzymes, suggesting improved metabolic capacity and liver function. The current study provided evidence for new dietary strategies based on matcha supplementation to ameliorate lipotoxicity-induced obesity and NALFD.

## 1. Introduction

Obesity is a severe, chronic, and progressive condition that has reached epidemic proportions globally. Many studies have confirmed the strong association between obesity and comorbidities such as hypertension, osteoarthritis, cardiovascular diseases, type 2 diabetes, and non-alcoholic fatty liver disease (NAFLD) [1]. The liver plays a key role in various aspects of lipid metabolism. Excessive intake of a high-fat diet (HFD) induces the overload of free fatty acids in the liver, leading to the steatosis and apoptosis of liver cells [2]. In the subsequent process, there will be hepatic inflammation and fibrosis, and may further progress to cirrhosis, liver failure, and liver cancer [3]. Since the related metabolic syndrome is a complex disease caused by a dynamic interaction between genetics and environmental factors, no effective medical interventions can treat across the full range [4]. Thus, an option for obesity and related NAFLD with good curative efficacy and fewer side effects is an unmet medical need. In recent years, natural antioxidants, particularly treated through dietary interventions, became widely applied currently [5,6,7].

Green tea is a worldwide consumed nonalcoholic beverage. Many studies have confirmed the efficacy of green tea in maintaining health and reducing the risk of various chronic diseases [8,9,10]. However, some papers have pointed out that a high-dose of green tea catechins may cause liver adverse reactions [11,12,13]. Therefore, it is important to clarify the toxicological effects of green tea and its extracts on the liver and related molecular mechanisms. Matcha is finely-milled green tea made from shaded young tea leaves and processed by low-temperature grinding [14,15], which has been widely used as a health-beneficial food additive in cookies, candies, chocolates, ice cream, puddings, etc. [16]. Compared to green tea, matcha excels in high contents of chlorophyll and catechin due to the tea leaves been protected from direct sunlight before harvest [17]. Moreover, with a different intake method from traditional green tea, both water-soluble and water-insoluble ingredients in matcha can be ingested and utilized. Previous studies suggested that matcha plays an important role as an antioxidant, anticarcinogen, anti-inflammatory, and anti-hypercholesterolemia [18,19,20,21]. More relevantly, dietary matcha supplement could inhibit lipid accumulation and ameliorate metabolic damage in obese mice induced by HFD [22,23]. Matcha is a potential intervention against obesity and related NAFLD. On this basis, the underlying NAFLD restraint mechanisms of matcha remain to be fully elucidated.

In this study, we carried out an in vivo experiment through an HFD-induced obese mouse model to evaluate the effects of matcha on alimentary obesity and related metabolic disorders. Liver transcriptome analysis was involved in this study to reveal the possible molecular mechanisms of those effects, which might relate to lipid metabolism alteration and hepatic cytochrome P450 enzymes-related inflammation regulation. Our results provide a novel insight into the anti-obesity mechanism of matcha green tea and potential dietary intervention for obesity and related metabolic syndrome.

## 2. Materials and Methods

### 2.1. Chemical Analysis of the Matcha Sample

A matcha sample was performed according to our previous experimental method [24] with some modifications. Briefly, powdered matcha green tea (10 g) was ultrasonically extracted twice with 150 mL of boiling distilled water in a boiling water bath for 30 min. The total infusion was concentrated using a vacuum rotary evaporator at 70 °C and the extract was freeze-dried using a freeze dryer (Christ, Osterode, Germany).

The total phenolic content assay was determined by the Folin–Ciocalteu method. The protein content assay was determined via the Coomassie brilliant blue method. Amino acid content assay was determined using the ninhydrin colorimetry method. The caffeine and tea catechins assay was determined using the Shimadzu LC-2010A HT HPLC system (Shimadzu Corporation, Tokyo, Japan). The above methods were implemented according to our previous report [25]. The total sugar content assay was determined by using the phonel-sulfate method according to Dubois’ report [26]. All chemical standards were of high-performance liquid chromatography grade.

### 2.2. Animals and Diet

All of the animal experiments were approved by the Animal Care and Use Committee at Zhejiang University (ethic approval code: ZJU20190065) and were performed following guidelines. In total, 25 C57BL/6 mice (male, 4-week-old) were obtained from Shanghai SLAC Laboratory Animal (Shanghai, China). The mice were maintained under controlled conditions with a temperature of 22 ± 1 °C, a constant humidity of 55 ± 5%, and a 12/12 h light/dark cycle. Feed and water were provided ad libitum during the experimental period. After a one-week-acclimation to the laboratory environment, the mice were randomly assigned to 5 groups (*n* = 5) and fed the following feeds for 6 weeks: a normal chow diet (NCD), a high-fat diet (HFD), and a high-fat diet blending with matcha (HML, HMM, HMH; with 0.1%, 0.5%, 1% matcha). The normal chow diet (10% energy from fat, #D12450J) and high-fat diet (60% energy from fat, #D12492) for mice were supplied by Research Diets, Inc. Co., Ltd. (New Brunswick, NJ, USA). Body weights and food intakes were recorded weekly. At the end of the sixth week, mice were sacrificed under isoflurane anesthesia.

### 2.3. Collection of Serum and Tissue Samples

Blood samples were collected from mice that were fasted for 12 h at the end of the sixth week. The serum biochemical analysis (including serum glucose, total triglyceride, cholesterol, low-density lipoprotein, and high-density lipoprotein) was determined by employing an automatic biochemical analyzer (TBA-40FR, Toshiba Medical, Tokyo, Japan). The epididymal white adipose tissue (eWAT), perirenal white adipose tissue (pWAT), subcutaneous white adipose tissue (sWAT), and liver tissue were collected and weighed, then immediately fixed in 4% formalin for H&E staining or otherwise stored at −80 °C for subsequent experiments.

### 2.4. H&E Staining

After the prior fixation for 24 h, the tissue samples were mounted in paraffin blocks, sliced at 5 μm, and stained with hematoxylin and eosin (H&E). The stained samples were detected with a microscope (Zeiss, Oberkochen, Germany).

### 2.5. Oil Red O Staining

Oil red O staining was performed with an oil red O stain kit (Solarbio, Beijing, China). The frozen sections of the liver tissues were dried and stained with oil red O according to the kit instructions. Images were obtained by a microscope (Zeiss, Oberkochen, Germany).

### 2.6. Enzyme-Linked Immunosorbent Assays (ELISA)

The concentrations of TNF-α, IL-6, and IL-1β in liver tissues were determined using an enzyme-linked immunosorbent assay (Kenuodi Biotechnology Co., Ltd., Quanzhou, China) according to the kit instructions.

### 2.7. Hepatic Transcriptome Sequencing and Annotation

Liver tissues from the NCD, HFD, and HMH mice at 6 weeks were prepared for RNA sequencing. Transcriptome sequencing (RNA-seq) was carried out by the BGI Life Science Research Institution (Shenzhen, China). Procedures for RNA preparation, library construction, and sequencing on the BGISEQ-500 platform were to refer to the previous report [27]. Standard bioinformatics analysis was performed by the BGI Life Science Research Institution. The enrichment analysis based on Kyoto Encyclopedia of Genes and Genomes (KEGG) was carried out for biological description of differentially expressed genes (DEGs).

### 2.8. Quantitative Real-Time Polymerase Chain Reaction (qRT-PCR) Analysis

RNA of hepatic tissues was prepared using the Trizol reagent according to the manufacturer’s protocol and our previous report [28]. Then, the RNA was reversely transcribed using a cDNA reverse transcription kit (Invitrogen, Carlsbad, CA, USA). Synthesized cDNA was amplified using the SYBR Green PCR Master Mix (Applied Biosystems, Foster City, CA, USA) on the LightCycler480 real-time system (Roche, Switzerland). The sequences of the primers in this study are listed in Table 1.

### 2.9. Statistical Analysis

All figures were plotted by GraphPad Prism Software version 8.0 (GraphPad Software Inc., San Diego, CA, USA), and statistical analyses were performed by SPSS 20.0 (IBM Corporation., Armonk, NY, USA). Data and results were expressed as mean ± standard error of the mean (SEM). The Student *t*-test was used for the analysis of data between two groups, and one-way analysis of variance (ANOVA) followed by Tukey’s multiple comparison post hoc was used for multiple comparisons. Differences were considered statistically significant when *p* < 0.05 and indicated with different superscript letters.

## 3. Results

### 3.1. Bioactive Compounds of the Matcha Sample

**Figure 1:**
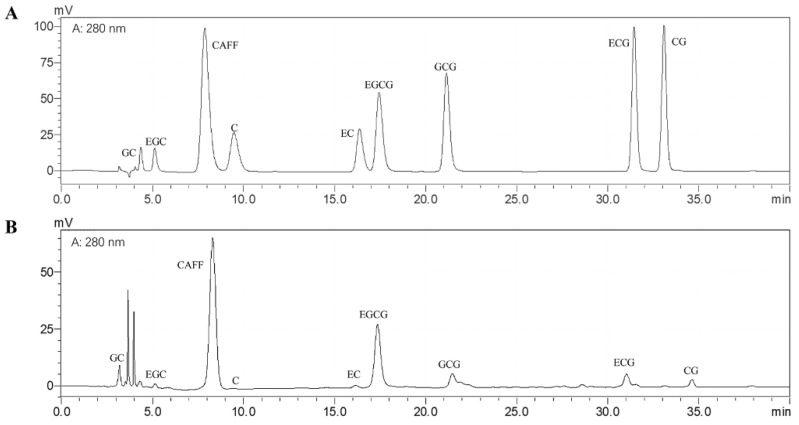
The UPLC chromatograms of catechin monomers and caffeine in the matcha sample. (**A**) Standard mixture; (**B**) matcha sample.

The major components of the matcha sample were evaluated and shown in Table 2. HPLC analysis was used to determine the amount of catechins monomers and caffeine (Figure 1). Results show that rich tea polyphenols and catechins are the important basis of the health benefits of matcha, which is consistent with our previous research results [22].

### 3.2. Matcha Ameliorated HFD-Induced Obesity

**Figure 2:**
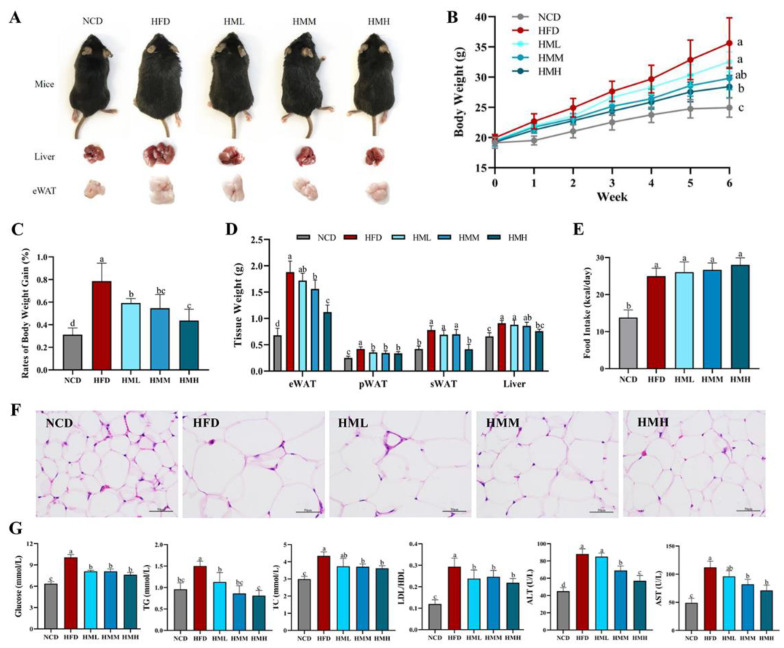
Effects of matcha on high-fat diet-induced obesity. (**A**) Representative images of the mice and tissues in different groups; (**B**) body weight evolution; (**C**) rates of body weight gain; (**D**) tissue weight; (**E**) food intake; (**F**) hematoxylin-eosin (H&E) staining of the epididymal adipose tissue; (**G**) serum biochemical parameters. Data are expressed as means ± SEM (*n* = 5). Means with different letters (a–d) were considered significantly different at *p* < 0.05 according to Tukey’s test. NCD, mice on a normal chow diet; HFD, mice on a high-fat diet; HML, mice on a high-fat diet with 0.1% matcha; HMM, mice on a high-fat diet with 0.5% matcha; HMH, mice on a high-fat diet with 1.0% matcha.

The effects of matcha on HFD-induced obesity were shown in Figure 2. A high-fat diet significantly increased the body weight and the tissue weight of eWAT, pWAT, sWAT, and liver, while the dietary supplement of matcha alleviated these obese conditions (Figure 2A–D) without affecting the energy intake (Figure 2E). A histological analysis showed that the size of adipocytes in the HFD group was much larger than that in the NCD group. The size of adipocytes decreased in varying degrees with the change of supplementary matcha concentration (Figure 2F). When considering the serum biochemical parameters, HFD mice showed hyperglycemia and hypercholesterolemia with higher levels of blood glucose, TG, TC, and an increased LDL/HDL ratio. Compared with the HFD group, the matcha-treated groups showed reduced levels of blood glucose, TG, TC, and LDL/HDL ratio in a dose-dependent manner, suggesting that matcha has the potential to regulate blood sugar and blood lipids (Figure 2G). Furthermore, the serum enzymes ALT and AST were considered as key biochemical indicators of hepatic injury degree [29]. As shown in Figure 2G, the activities of ALT and AST in the HFD mice were significantly higher than that of the NCD mice, while the dietary supplement of matcha caused the reduction of the serum ALT and AST activities in a dose-dependent manner.

### 3.3. Matcha Improved Hepatic Lipid Accumulation and Inflammation

**Figure 3:**
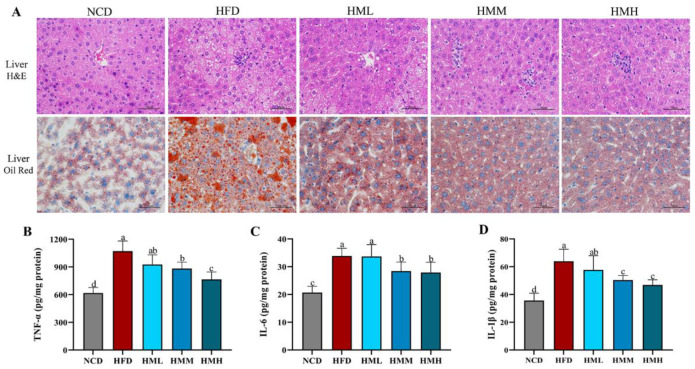
Effects of matcha on hepatic lipid infiltration and inflammation. (**A**) Morphology of liver sections stained with oil red O and hematoxylin-eosin (H&E) staining; (**B**–**D**) levels of major inflammatory cytokines (TNF-α, IL-6, and IL-1β) determined by ELISA. Data are expressed as means ± SEM (*n* = 5). Means with different letters (a–d) were considered significantly different at *p* < 0.05 according to Tukey’s test.

Histological analysis showed that excessive lipid accumulation and infiltration of inflammatory cells occurred in the liver tissues of HFD mice, suggesting chronic hepatocyte damage and inflammation; liver sections of mice fed with matcha showed very mild steatosis and inflammatory foci (Figure 3A). We further assessed the TNF-α, IL-6, and IL-1β amounts in liver tissues. The results revealed markedly increased amounts of these biomarkers in the HFD group in comparison with the NCD group; the matcha treatment markedly reversed these effects (Figure 3B–D), suggesting that the matcha treatment effectively ameliorated HFD-induced hepatic steatosis and inflammatory infiltration.

### 3.4. Matcha Induced Transcriptome Alterations in the Liver

**Figure 4:**
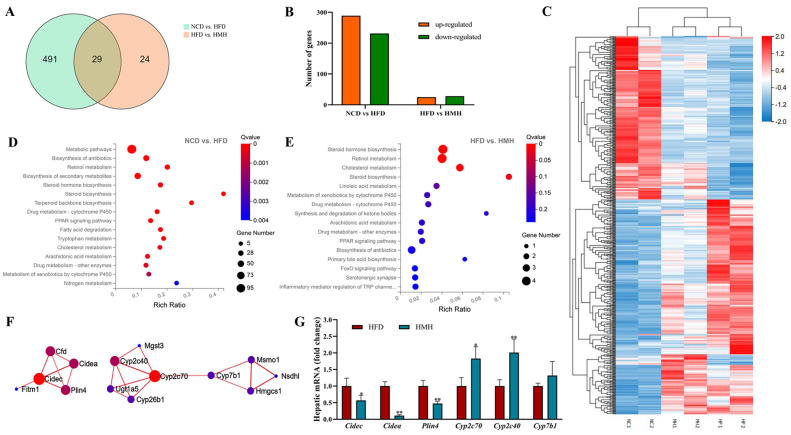
Effects of matcha on the liver transcriptome of mice. (**A**) Venn diagram analysis for co-expressed genes derived from RNA sequencing; (**B**) number of significantly up-regulated and down-regulated genes in comparisons of the NCD and HFD groups or HFD and HMH groups; (**C**) heatmap that shows the expression pattern of all DEGs and the hierarchical clustering of the liver samples based on the transcriptomic profiles. Red indicates upregulation, blue indicates downregulation; (**D**,**E**) KEGG pathway analysis of differentially expressed genes between the NCD and HFD groups or HFD and HMH groups; (**F**) *Cidec* and *Cyp2c70* related molecular networks—the redder the color, the more significant it is; (**G**) quantitative real-time PCR of *Cidec*-related genes and *Cyp2c70*-related genes. Data are expressed as means ± SEM (n = 3). * *p* < 0.05, ** *p* < 0.01 compare to the HFD group.

Transcripts of hepatic DEGs were characterized using RNA-seq analyses on the basis of the observed hepatic steatosis and inflammatory infiltration induced with an HFD. Intersections of DEGs in the liver tissues between the NCD group and HFD group (NCD vs. HFD), as well as between the matcha-treated group and HFD group (HFD vs. HMH) were shown in Figure 4A as a Venn diagram. Compared with the NCD group, the HFD group resulted in 520 DEGs (289 upregulated; 231 downregulated); the HMH group altered the expression of 53 DEGs (25 upregulated; 28 downregulated) compared to the HFD group (Figure 4B). A heat map analysis showed that, compared with the NCD group, the upregulated DEGs in the HFD group were somehow downregulated by matcha treatment. While the downregulated DEGs in the HFD group were partly upregulated by matcha treatment (Figure 4C).

According to the KEGG annotation and the official classification, signaling pathway enrichment analysis was further evaluated. Figure 4D,E present the KEGG pathways in the top 15 enrichment levels of DEGs in the NCD vs. HFD and the HFD vs. HMH, respectively. In differentially expressed genes which were identified by comparison of the control group with the HFD group, we found that a high-fat diet causes a series of metabolic disorders, the main signaling pathways were related to lipid metabolism, endocrine regulation and amino acid metabolism (Figure 4D). While matcha treatment could reverse several signaling pathways related to lipid metabolisms, such as PPAR signaling pathway, cholesterol metabolism, and linoleic acid metabolism. It may be associated with the decreased blood lipid level and remitted pathological process of fatty liver, proving evidence of the efficacy of matcha in the treatment of NAFLD. Furthermore, most of the up-regulated genes in the matcha-treated group were found to be involved with the recovery of liver function, such as retinol metabolism, metabolism of xenobiotics by cytochrome P450, and drug metabolism (cytochrome) P450 (Figure 4E).

To explore the mechanism of matcha alleviates NAFLD, we selected the genes changed by HFD and reversed by the matcha treatment for PPI network analysis. The constructed PPI network (Figure 4F) showed that genes were mainly enriched in lipid droplet-associated genes (with *Cidec* as the core gene) and cytochrome P450-associated genes (with *Cyp2c70* as the core gene). Furthermore, we conducted quantitative real-time PCR with the key genes *Cidec*, *Cidea*, *Plin4*, *Cyp2c70*, *Cyp2c40,* and *Cyp7b1*. Results showed that the matcha treatment indeed reversed the expression of the *Cidec*-related genes and *Cyp2c70*-related genes (Figure 4G), suggesting that these pathways may play leading roles in the protective effects of matcha on the HFD-induced hepatotoxicity.

## 4. Discussion

A lot of research shows that excessive consumption of high-calorie foods has induced the prevalence of obesity and related NAFLD [2]. NAFLD represents a disease spectrum that ranges from steatosis to nonalcoholic steatohepatitis, liver cirrhosis, and hepatocarcinoma [3]. Although the preventive effects of green tea against NAFLD are well established in animal experiments and epidemiological studies, the underlying mechanisms remain to be ascertained [30]. This research takes matcha, a new type of green tea popular in recent years, as the experimental object to examine its effects in preventing obesity and related NAFLD. Our study demonstrated that matcha green tea could improve the pathological state of HFD-induced obesity and related liver dysfunction. After a high-fat diet for six weeks, the mice in the HFD group showed a series of symptoms of significant weight gain, excess adipose tissue mass, hepatic lipid deposition, and impaired liver function. While matcha green tea could effectively inhibit lipid accumulation, decrease hyperglycemia, improve dyslipidemia, and decrease ALT and AST levels in obese C57BL/6 mice. Furthermore, the levels of major inflammatory cytokines (TNF-α, IL-6, and IL-1β) associated with hepatic inflammation were also significantly lower. These results revealed that matcha green tea could remit the pathological process in obesity-induced hepatic steatosis and steatohepatitis.

The RNA-seq analysis showed that a high-fat diet resulted in wide modification of liver transcriptome architecture with up to 520 differentially regulated transcripts in comparison to the mice fed with a normal diet. The main function categories such as lipid metabolism, endocrine regulation, and amino acid metabolism were enriched significantly from the DEGs between the HFD group and NCD group. Even though matcha effectively reversed the symptoms of NAFLD, far fewer DEGs were modulated by its dietary supplementation, i.e., 53 transcripts. In these pathways including the PPAR signaling pathway, retinol metabolism and cytochrome P450 were affected significantly by matcha treatment. The pathway analysis and PPI network revealed the involvement of a string of genes, mainly including *Cidec*-related genes and *Cyp2c70*-related genes, which were down- and up-regulated in the liver tissues of the matcha-treated group, respectively. Then, these targets were confirmed via RT-PCR analysis.

NAFLD is believed to result from the excessive accumulation of triglyceride-rich lipid droplets in the liver [31]. Intracellular lipid droplets are coated with various lipid droplets-associated proteins (LDAPs) in the CIDE (cell death-inducing DNA fragmentation factor-α-like effector) family and PLIN (perilipin) family, which regulate lipid droplet synthesis and hepatic lipid homeostasis [32,33]. CIDE family proteins are key players in lipid metabolism and the control of NAFLD progression. For example, The CIDEA localizes on the surface of lipid droplets and can promote hepatic steatosis by sensing dietary fatty acids [34]. It is related to the augmented hepatic lipid accumulation during the development of NAFLD. Zhou et al. found that the knockdown of *Cidea* in *ob/ob* mice resulted in significantly reduced hepatic lipid accumulation and alleviated hepatic steatosis [35]. Similarly, CIDEC, also known as fat-specific protein 27 (FSP27) in mice, contributes to triglyceride accumulation and is found to be highly expressed in fatty liver [34]. Xu et al. have reported that the sustained CIDEC elevation in hepatocytes could cause chronic liver injury and inflammation [36]. PLIN (includes 5 members, PLIN1 to PLIN5) is a family of LDAPs found predominantly in white adipose tissue, with the effect of promoting lipid droplet formation by regulating the hydrolysis of stored triglyceride [32]. From the current study, the focus is mainly on the role of PLIN2 in liver steatosis [37,38], whereas our results bring to light the potential of PLIN4. PLIN4 has a molecular weight of nearly three-fold that of other PLINs, which was first identified as an adipocyte-specific protein induced during adipocyte differentiation [39]. It is expressed predominantly in white adipose tissue, yet few effects have been studied specifically in hepatocytes [40]. Nunn et al. observed an upregulation of *Plin4* in free fatty acid overload HepaRG cells, providing a mechanistic link between *Plin4* and hepatic lipid storage mechanisms. Herein, we found that matcha suppressed the serum lipid level and the lipid accumulation in liver and adipose tissue, which might be related to the down-regulation of *Cidec*, *Cidea*, *Plin4,* and other related genes.

Obesity often induces a high prevalence of metabolic syndrome, which is closely associated with a systemic status of chronic low-grade inflammation [41]. Cytochrome P450 monooxygenase enzymes (P450s or CYPs) is a superfamily of heme-containing enzymes abundant in the liver [42]. They are crucial to the metabolism of endogenous substances, including steroids and bile acids, as well as exogenous substances, including drugs, natural compounds, carcinogens, and environmental chemicals [43]. Inflammatory stimuli are shown to impact the metabolism of endogenous and exogenous substances through the downregulation of hepatic CYPs, which is a vital pathogenesis of liver function disorders and severe liver diseases [44]. In the present study, some CYP2C family members were possible targets of matcha administration. The CYP2C subfamily is the key enzymatic system in the metabolism of arachidonic acid to bioactive eicosanoids [45]. CYP2C-derived eicosanoids have essential functions in regulating immune and inflammatory responses [24]. Kim et al. reported a reduced CYP2C catalytic activity in chemically induced diabetic rats [46]. Sarah et al. found that hepatic CYP2C metabolic activities significantly decreased in diet-induced obese mice [47]. Our study, in agreement with these findings, suggested that CYP2C activities, particularly *Cyp2c70* and *Cyp2c40*, were impaired by HFD. Pass and coworkers found a down-regulation of *Cyp2c40* in diabetic mice, which seemed to be mediated by the peroxisome proliferator-activated receptor-α. Honda et al. generated *Cyp2c70* KO mice using the CRISPR-Cas9 system and observed high expression levels of inflammatory cytokines *Il1b* and *Tnfa*, and notable infiltration of lymphocytes and neutrophils in the liver, implying that *Cyp2c70* might be involved in the regulation of hepatic inflammatory response [48]. Our findings are consistent with these studies that matcha improved the release of inflammatory cytokines and declined the expression of *Cyp2c70*-related genes in the liver induced by HFD. Until now, research on the effects of tea on the regulation of CYPs mainly focuses on CYP2E1 [49], while our study indicates the *cyp2c* subfamily as a promising target for matcha green tea to improve steatosis hepatitis.

## 5. Conclusions

**Figure 5:**
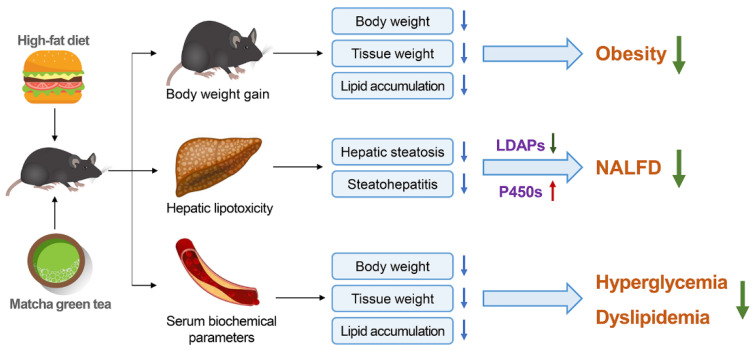
Schematic diagram showing the possible mechanisms of matcha green tea preventing high-fat diet-induced obesity and hepatic lipotoxicity.

Taken together, HFD induced obvious symptoms of obesity and hepatic lipid accumulation, and the resultant lipotoxicity promotes NAFLD progression. Integrated analysis of the biochemical parameters, histopathology, liver transcriptome, and gene expression revealed that matcha green tea was a natural dietary/nutraceutical source to prevent lipotoxicity-induced obesity and liver injury. We found that matcha reduced the serum levels of glucose, TG, and TC, as well as ALT and AST activities in C57BL/6 mice fed a HFD. At the same time, matcha also significantly improved obesity-related lipid accumulation and steatosis hepatitis. Fundamentally, downregulation on expression levels of LDAPs, such as *Cidec*, *Cidea*, and *Plin4*, suggests that the inhibitory effect of matcha on lipid accumulation may go through this pathway. Moreover, activation of the *cyp2c* subfamily can reduce inflammation and improve liver metabolism, which is also a potential mechanism underlying the beneficial effects of matcha on hepatic lipotoxicity (Figure 5).

The results provided evidence for new dietary strategies based on matcha supplementation to ameliorate HFD-induced obesity and NALFD. Nevertheless, how the critical genes in the pathways related to lipid metabolism and inflammatory responses interact with each other is still deficient. Further investigation is required to explore the molecular mechanism of matcha on the precise crosstalk between lipid homeostasis and CYP-involved hepatic biotransformation function.

## Figures and Tables

**Table 1:** Primers of RT-PCR.

Gene	Forward Primer (5′ to 3′)	Reverse Primer (5′ to 3′)
*Cyp2c70*	TATGGGCTTTTGCTCCTGCT	GGTCTCCGATGTCTACCAATCAC
*Cyp2c40*	GCTCACCCTGTGATCCCCAATTCA	TTGAGAAAAACAGCATAGCAG
*Cyp7b1*	AATTGGACAGCTTGGTCTGC	TTCTCGGATGATGCTGGAGT
*Cidec*	GTGTCCACTTGTGCCGTCTT	CTCGCTTGGTTGTCTTGATT
*Cidea*	TCCTCGGCTGTCTCAATG	TGGCTGCTCTTCTGTATCG
*Plin4*	GCAGTATCTGGAGGTGTGATG	TGTGTCCTTCGTATTGGTGAG
*Gapdh*	CCTCGTCCCGTAGACAAAATG	TGAGGTCAATGAAGGGGTCGT

**Table 2:** Chemical analysis of the matcha sample.

Compounds (%)	Matcha
Tea polyphenols	19.84 ± 0.67
Amino acid	4.19 ± 0.01
Total sugars	3.14 ± 0.15
Protein	1.15 ± 0.10
GC	1.83 ± 0.18
EGC	1.15 ± 0.09
C	0.08 ± 0.11
EC	0.44 ± 0.02
EGCG	7.02 ± 0.93
GCG	1.27 ± 0.20
ECG	0.83 ± 0.13
CG	0.09 ± 0.02
Caffeine	6.58 ± 0.01

GC, (+)-gallocatechin; EGC, (−)-epigallocatechin; C, (+)-catechin; EC, (−)-epicatechin; EGCG, (−)-epigallocatechin gallate; GCG, (+)-gallocatechin gallate; ECG, (−)-epicatechin gallate; CG, (−)-epigallocatechin gallate.

## Data Availability

The data presented in this study are available on request from the corresponding author. The data are not publicly available due to the data also forms part of an ongoing study.
